# Expression and prognostic value of FKBP51 in Hodgkin lymphoma

**DOI:** 10.3389/fimmu.2025.1604920

**Published:** 2025-11-03

**Authors:** Silvia Varricchio, Simona Romano, Daniela Russo, Antonio Travaglino, Rosaria Cappiello, Mariarosaria Cervasio, Gennaro Ilardi, Laura Marrone, Fabrizio Pane, Marco Picardi, Marcello Persico, Elena Vigliar, Gennaro Acanfora, Maria Fiammetta Romano, Massimo Mascolo

**Affiliations:** ^1^ Department of Advanced Biomedical Sciences, Pathology Section, University of Naples “Federico II”, Naples, Italy; ^2^ Department of Molecular Medicine and Medical Biotechnology, University of Naples Federico II, Naples, Italy; ^3^ Unit of Pathology, Department of Medicine and Technological Innovation, University of Insubria, Varese, Italy; ^4^ Department of Clinical Medicine and Surgery, Hematology Section, University of Naples “Federico II”, Naples, Italy; ^5^ Department of Clinical Medicine and Surgery, University of Naples “Federico II”, Naples, Italy; ^6^ Department of Public Health, University of Naples “Federico II”, Naples, Italy

**Keywords:** Hodgkin lymphoma, FKBP51, Bc2, tumor microenvironment, prognosis

## Abstract

**Background:**

Hodgkin lymphoma (HL) is characterized by rare Hodgkin/Reed-Sternberg (H/RS) cells surrounded by a predominant immune infiltrate that shapes tumor biology and influences prognosis. FKBP51, an immunophilin and NF-κB/Akt modulator, is implicated in cancer progression, but its role within the HL tumor microenvironment (TME) remains unclear.

**Methods:**

We retrospectively analyzed 103 HL cases by immunohistochemistry for FKBP51, Bcl2, and immune subsets (CD4, CD8, CD68, CD163), with quantitative PCR of *FKBP5, TRAF2, PCNA, XIAP*, and *BCL2* in 36 cases. Spatial immune architecture was assessed morphologically and via image analysis, with a focus on CD4 T-cell rosettes. Prognostic associations were evaluated through multivariate analyses.

**Results:**

FKBP51 was detected in both H/RS cells and infiltrating lymphocytes. While nuclear FKBP51 in H/RS cells lacked prognostic significance, FKBP51 expression in the TME correlated with adverse outcomes and remained an independent prognostic factor. CD4 T cells were the predominant immune cell subset and the main FKBP51-positive population. However, it was the density of tumor-associated macrophages (TAMs), rather than CD4 T-cell density, that held prognostic significance. CD4 T cells frequently formed rosette-like structures around H/RS cells, a spatial organization associated with the highest FKBP51 scores and unfavorable prognosis. CD8 T cells were less abundant, increased in advanced stages along with TAMs, and exhibited limited FKBP51 expression. Gene expression analysis showed FKBP51 correlation with proliferative and anti-apoptotic transcripts (*PCNA, TRAF2, XIAP*), supporting a protumor, NF-κB-driven microenvironment.

**Conclusions:**

FKBP51 expression in CD4 tumor-infiltrating lymphocytes, rather than tumor cells, defines a protumor TME that supports H/RS cell survival. Spatial immune architecture, particularly CD4 rosettes and TAM density, holds prognostic relevance in HL. FKBP51 may serve as a biomarker for risk stratification.

## 1 Introduction

Hodgkin lymphoma (HL) is one of the most common lymphomas in the Western world, accounting for about half of all lymphomas in children and young adults (1). The first-line treatment for classic HL consists of ABVD (doxorubicin, bleomycin, vinblastine, and dacarbazine) polychemotherapy with or without radiotherapy (2). The number of first-line cycles and radiation dose leading to a complete response in most patients is determined by prognostic factors such as extra-nodal disease, mediastinal bulky, high erythrocyte sedimentation rate and B symptoms (3–5). A significant number of patients with HL experience refractory disease or relapse even after achieving a complete response to therapy. In cases of relapse or refractory HL, immunotherapy options such as PD-1 inhibitors (6) or the CD30 inhibitor brentuximab vedotin (7) have demonstrated efficacy, however, achieving a definitive cure in such cases remains challenging (6, 7). Identifying prognostic biomarkers linked to inadequate responses in HL can help pathologists deliver more accurate diagnostic definitions and characterizations, aid in patient stratification, and discover new therapeutic targets.

Diagnosing HL presents several challenges for pathologists, H/RS cells are generally rare in tissue samples, making them difficult to detect. HL is characterized by large-sized neoplastic B cells, termed “Hodgkin cells” and “Reed-Sternberg cells” (H/RS cells), scattered in a dominant background of non-neoplastic immune cells. Such microenvironments comprise T and B lymphocytes, eosinophils, and macrophages in classic HL (which accounts for >95% of all HL cases) and mainly lymphocyte in nodular lymphocyte-predominant HL (8).

H/RS cells secrete chemokines that attract CD4 T cells and Tregs while repelling CD8 T cells (9, 10). A distinctive feature of HL is the formation of “rosettes,” where CD4 T lymphocytes surround H/RS cells. This unique organization of immune cells is driven by a combination of chemokine signaling, receptor-ligand interactions, and other immune evasion mechanisms (11). Instead of initiating an anti-tumor response, CD4 T cells are co-opted by H/RS cells to provide growth signals, promote immune suppression, and shield the tumor from cytotoxic effects (9, 10, 12). This relationship highlights a complex interaction within the HL TME, where CD4 T cells not only fail to eliminate H/RS cells but also support their survival.

In HL, a deregulated NF-κB pathway represents one of the key mechanisms promoting survival of H/RS cells (13). FKBP51 is an immunophilin and cochaperone that plays a crucial role in NF-κB activation (14). It exerts an essential role in supporting lymphocyte activation and promoting their proliferation (15). Furthermore, numerous studies have reported abnormal FKBP51 expression in various cancers (16–20) and have proposed it as a reliable prognostic marker in human solid tumors, including mycosis fungoides (21).

FKBP51 has not yet been studied in HL. This research aims to evaluate its expression and prognostic significance alongside Bcl-2, which is a well-known prognostic marker for HL (22). Additionally, the study will examine any potential association between FKBP51, and the gene expression of molecular factors involved in proliferation and anti-apoptosis that are under the transcriptional control of NF-κB.

## 2 Materials and methods

### 2.1 Case selection

Clinical data and formalin-fixed, paraffin-embedded (FFPE) tissue blocks of 103 consecutive patients with HL, diagnosed between January 2012 and February 2020, were retrieved from the archives of the Hematology Unit and Pathology Unit of the Federico II University of Naples. Three expert pathologists (AT, DR and MM) re-evaluated the histological slides to confirm the diagnosis.

The study complies with the Institutional Ethics Committee guidelines, which, following Italian law, do not ask for the Ethical Committee approval on the current research topics.

Furthermore, it follows the Declaration of Helsinki, for studies based only on retrospective analyses on routine FFPE tissue, for which written informed consent from the living patient is required, following the indication of Legislative Decree no. 196/03 (Codex on Privacy) at the time of the intervention for the CL.

### 2.2 Quantitative PCR

Total RNA extraction was performed on 36 tissue samples by using a FFPE RNA Purification Kit (Merck, Darmstadt, Germany), according to the manufacturer’s instructions and as previously described (21). One μg of each RNA was used for cDNA synthesis with iScriptTM Reverse Transcription (Bio-Rad, Hercules, CA, USA). Gene expression was quantified by qPCR using SsoAdvancedTM SYBR Green Supermix (Bio-Rad) and specific qPCR primers were used for the relative quantitation of the transcripts, performed using co-amplified 18S as an internal control for normalization. To represent the expression differences between different patients, relative quantification of transcripts was calculated with the 2^–ΔΔCt^ method by choosing as reference samples the average 2^–ΔΔCt^ values obtained from peripheral blood mononuclear cells (PBMCs) of 3 healthy donors. For FKBP51, XIAP and 18S specific real-time-validated QuantiTect primers from Qiagen (Valencia, CA, USA) were used. Oligo sequences of TRAF2 and BCL2 were previously reported (14); oligo sequences for PCNA and P53 are indicated: hPCNA_Fw: 5’-CTGCAGAGCATGGACTCGTC-3’, hPCNA_Rev: 5’-GTAGGTGTCGAAGCCCTCAGA; hP53_Fw: 5’-ATCCTCACCATCATCACACTGG-3’, hP53_Rev: 5’-TCTTGCGGAGATTCTCTTCCTC-3’.

### 2.3 Immunohistochemistry

For each case, we selected a block of tissue fixed in formalin and embedded in paraffin with the highest abundance of H/RS cells and used it to obtain serial sections. Serial 4 µm tissue sections were cut from the paraffin blocks using an ordinary microtome and were mounted on TOMO^®^ IHC Adhesive Glass Slides (Matsunami Glass Ind., Ltd., Japan), for the immunohistochemical evaluation of FKBP51 (clone H-100, sc-13983 Santa Cruz Biotechnology; 1: 200 dilution), Bcl2 (clone SP66, 790–4604 Roche Diagnostics; prediluted), CD4 (clone SP35, 790–4423 Roche Diagnostics; prediluted), CD8 (clone SP239, 790–7176 Roche Diagnostics; prediluted), CD68 (clone KP1, 790–2931 Cell Marque; prediluted) and CD163 (clone MRQ26, 760–4437 Roche Diagnostics; prediluted). Immunohistochemical staining was performed on a Ventana Benchmark Ultra (Ventana Medical Systems Inc., Tucson, AZ) following previously described methods (21). The immunohistochemical expression was assessed through a combined score that considered both intensity and distribution of immunostaining. The intensity of immunostaining was categorized as follows: 0 = null; 1 = weak intensity; 2 = moderate intensity; 3 = strong intensity. The distribution of immunostaining was categorized as follows: 0=<10% of cells, 1 = 10-49%, 2=≥50%. The combined score was calculated as intensity + distribution, with a resulting score of 0-5. All immunohistochemical slides were evaluated by three expert pathologists (AT, DR and MM) after being blinded to the clinical data.

### 2.4 Statistical analyses

The association among immunohistochemical markers and between qPCR data (Prism GraphPad 7.0a for Macintosh) was assessed by using the Pearson correlation coefficient. The association among immunohistochemical markers was assessed by using the Pearson correlation coefficient. The association between the markers and oncologic outcomes were assessed by using univariate and multivariate binary logistic regression. A significant p-value ≤ 0.05 was adopted. Statistical analyses were performed by using the Statistical Package for Social Science (SPSS) 18.0 package (SPSS Inc., Chicago, IL, USA) and Prism GraphPad 7.0a for Macintosh.

## 3 Results

### 3.1 Clinical pathological data

The mean age of the 103 patients was 41 years (range 12-85); 49 patients were male, and 54 were female. All patients underwent Ann Arbor staging system with whole body FDG/PET scans and were treated accordingly with frontline ABVD. Information about the stage was available for 102 patients, out of which 4 were at stage I, 53 were at stage II, 17 were at stage III, and 28 were at stage IV. The mean follow-up was 121.3 months (range 1-156); during the observation time, 29 (28,2%) patients were lost at the follow-up, and so, their data were censored from the statistical analysis; 61 (59,2%) patients were alive with no evidence of disease at the last follow-up, while 7 (6,8%) died from disease and 6 (5,8%) had primary refractory disease. **Table 1** summarizes clinical-pathological data.

**Table 1:** Characteristics of the patients and immunohistochemical expression of FKBP51 and BCL2 in HL.

AGE, mean	41 y (range 12-85)
SEX, n	
- male - female	49 (47.6%) 54 (52.4%)
STAGE, n	
- I - II - III - IV	4 (3.9%) 53 (51.5%) 17 (16.5%) 28 (27.2%)
FOLLOW-UP, mean	121.3 m (range 1-156)
Bcl2 (H/RS cells) - 0 - 1 - 2 - 3 - 4 - 5	49 (47.6%) 10 (9.7%) 11 (10.7%) 14 (13.6%) 15 (14.6%) 4 (3.9%)
Bcl2 (background)*	
-2 -3 -4 -5	2 (1.9%) 38 (36.9%) 26 (25.2%) 35 (34%)
FKBP51 (H/RS cells)	
- 0 - 1 - 2 - 3 - 4 - 5	31 (30.1%) 17 (16.5%) 27 (26.2%) 9 (8.7%) 8 (7.8%) 11 (10.7%)
FKBP51 (background)	
- 1 - 2 - 3 - 4 - 5	21 (20.4%) 12 (11.7%) 25 (24.3%) 14 (13.6%) 31 (30.1%)

* IHC evaluation was not possible in two cases.

### 3.2 Immunohistochemical staining reveals background lymphocytes exhibiting strong and prominent FKBP51

**Figure 1:**
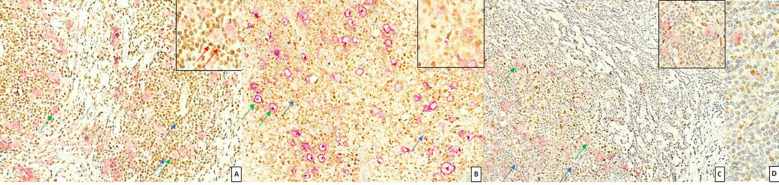
Expression of FKBP51 in Hodgkin lymphoma. **(A)** Double immunostaining for CD30 (red, green arrow) and FKBP51 (brown, blue arrow) (magnification 200X): FKBP51 negative in Hodgkin/Reed-Sternberg cells and strong (red arrow in inset) background lymphocytes in a poor prognosis HL case; **(B)** FKBP51 negative in Hodgkin/Reed-Sternberg cells and moderate in the background lymphocytes in a favorable prognosis HL case (magnification 200X). **(C)** FKBP51 negative in Hodgkin/Reed-Sternberg cells and weak in the background lymphocytes in a favorable prognosis HL case; **(D)** The blue color from hematoxylin staining indicates the cell nuclei negative for markers (magnification 400X).

**Figure 2:**
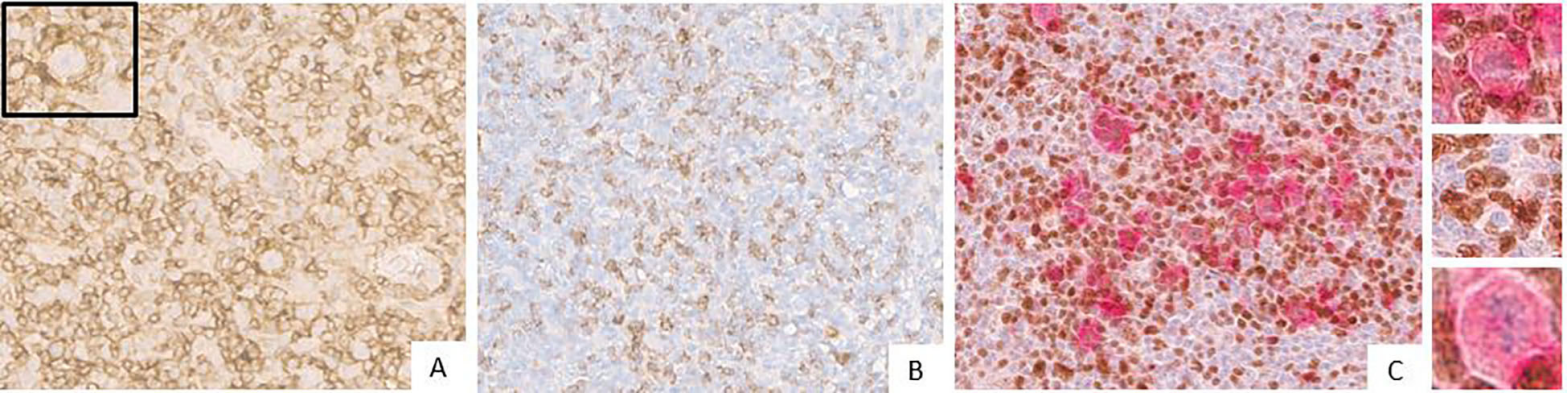
**(A)** Expression of CD4 (brown) in Hodgkin lymphoma (the inset displays Reed-Sternberg cell surrounded by a rosette of CD4 cells). The blue coloration reflects hematoxylin-stained nuclei in regions lacking marker expression. **(B)** Expression of CD 8 (brown) in Hodgkin lymphoma. The blue coloration reflects hematoxylin-stained nuclei in regions lacking marker expression. **(C)** Double immunostaining for CD30 (red) and FKBP51 (brown) in Reed–Sternberg cells. The insets show: (upper) a CD30-positive Reed–Sternberg cell surrounded by a rosette of CD4-positive lymphocytes; (intermediate) FKBP51-positive lymphocytes; and (lower) a CD30-positive H/RS cell. The blue coloration reflects hematoxylin-stained nuclei in regions lacking marker expression.

**Figure 3:**
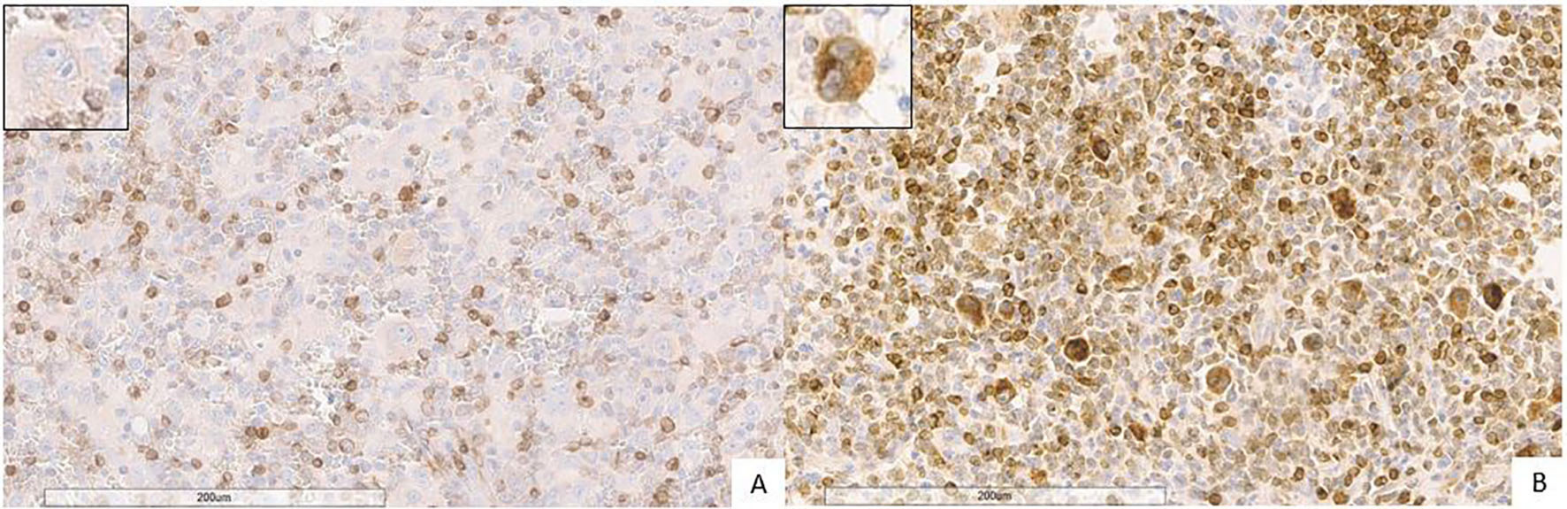
Expression of Bcl2 (brown) in Hodgkin lymphoma (magnification 20X). **(A)** Negative Bcl2 expression in Hodgkin/Reed-Sternberg cells. The blue coloration reflects hematoxylin-stained nuclei in regions lacking marker expression. **(B)** Strong Bcl2 expression in Hodgkin/Reed-Sternberg cells (the inset displays Hodgkin/Reed-Sternberg cell). The blue coloration reflects hematoxylin-stained nuclei in regions lacking marker expression.

FKBP51 expression in the H/RS cells was almost exclusively nuclear (**Figure 1**) and completely negative in 31 cases (30.1%). Most of the positive cases had weak and/or focal expression with a score 1 (n=17, 16.5%) or 2 (n=26, 26.2%); the remaining cases showed a score of 3 (n=9, 8.7%), 4 (n=8, 7.8%) or 5 (n=11, 10.7%) (**Table 1**). All HL cases showed a nuclear FKBP51 score ≥1 in the background lymphocytes; the expression was strongest in the lymphocytes adjacent to H/RS cells and showed at least moderate intensity in most cases, with a score of 4 (n=14, 13.6%) or 5 (n=31, 30.1%); the remaining cases showed scores 1 (n=21, 20.4%), 2 (n=12, 11.7%) or 3 (n=25, 24.3%) (**Figure 1**). The lymphocytes expressing FKBP51 appeared to be T-helper (CD4+ on immunohistochemistry) (**Figure 2**). There was a significant correlation between the expression of FKBP51 in H/RS cells and in the background lymphocytes (p=0.008) (**Table 2**). **Supplementary Figure S1** shows single IHC performed on consecutive serial sections, in parallel with the double IHC, using the conventional DAB chromogen (brown). The expression of Bcl2 in H/RS cells was cytoplasmic (**Figure 3**) and showed the following distribution of scores: 0, (complete negativity) in 49 cases (47.6%); score 1, in 10 cases (9.7%); score 2, in 11 cases (10.7%); score 3, in 14 cases (13.6%); score 4, in 15 cases (14.6%); and score 5, in 4 cases (3.9%). The expression of Bcl2 in the background lymphocytes showed scores 2 (n=2, 1.9%), 3 (n=38, 36.9%), 4 (n=26, 25.2%) or 5 (n=35, 34%). No correlation was found between H/RS Bcl2 expression and FKBP51 expression in either H/RS cells or in the background lymphocytes (**Table 2**).

**Table 2:** Correlation between immunohistochemical markers.

Biomarker	Bcl2 (H/RS cells)	Bcl2 (background)	FKBP51 (H/RS cells)	FKBP51 (background)
Bcl2 (H/RS cells)	–	p=0.725	p=0.337	p=0.961
Bcl2 (background)	p=0.725	–	p=0.490	p=0.475
FKBP51 (H/RS cells)	p=0.337	p=0.490	–	p=0.008*
FKBP51 (background)	p=0.961	p=0.475	p=0.008*	–

H/RS, Hodgkin/Reed-Sternberg; *: significant p-value.

### 3.3 Prognostic analysis reveals FKBP51 expression in the TME as an unfavorable factor for HL

A previous study identified Bcl-2 as a prognostic marker in Hodgkin lymphoma (HL) (22). In this study, we confirm that Bcl-2 expression in H/RS cells is associated with an unfavorable clinical outcome, with a p-value of 0.042 (**Table 3**). Furthermore, we have identified FKBP51 expression in the background lymphocytes as an additional prognostic marker, which shows a statistically significant p-value of 0.008 (**Table 3**). This significance was maintained in multivariate analysis for both Bcl-2 (p=0.035) and FKBP51 (p=0.008) (**Table 3**). In contrast, FKBP51 expression in H/RS cells and Bcl-2 in H/RS cells did not correlate with clinical outcomes, showing a p-value of 0.595 and 0.966, respectively (**Table 3**). Supplementary information, **Supplementary Figure S2** shows Kaplan–Meier survival curves based on H/RS Bcl-2 and background FKBP51 expression.

**Table 3:** Association between immunohistochemical markers and unfavorable outcome (refractoriness/relapse).

Biomarker	Univariate	Multivariate
Bcl2 (H/RS cells)	p=0.042*	p=0.035*
Bcl2 (background)	p=0.996	p=0.934
FKBP51 (H/RS cells)	p=0.595	p=0.566
FKBP51 (background	p=0.008*	p=0.008*

H/RS, Hodgkin/Reed-Sternberg; *: significant p-value.

### 3.4 Immunohistochemical characterization of inflammatory infiltrate

**Figure 4:**
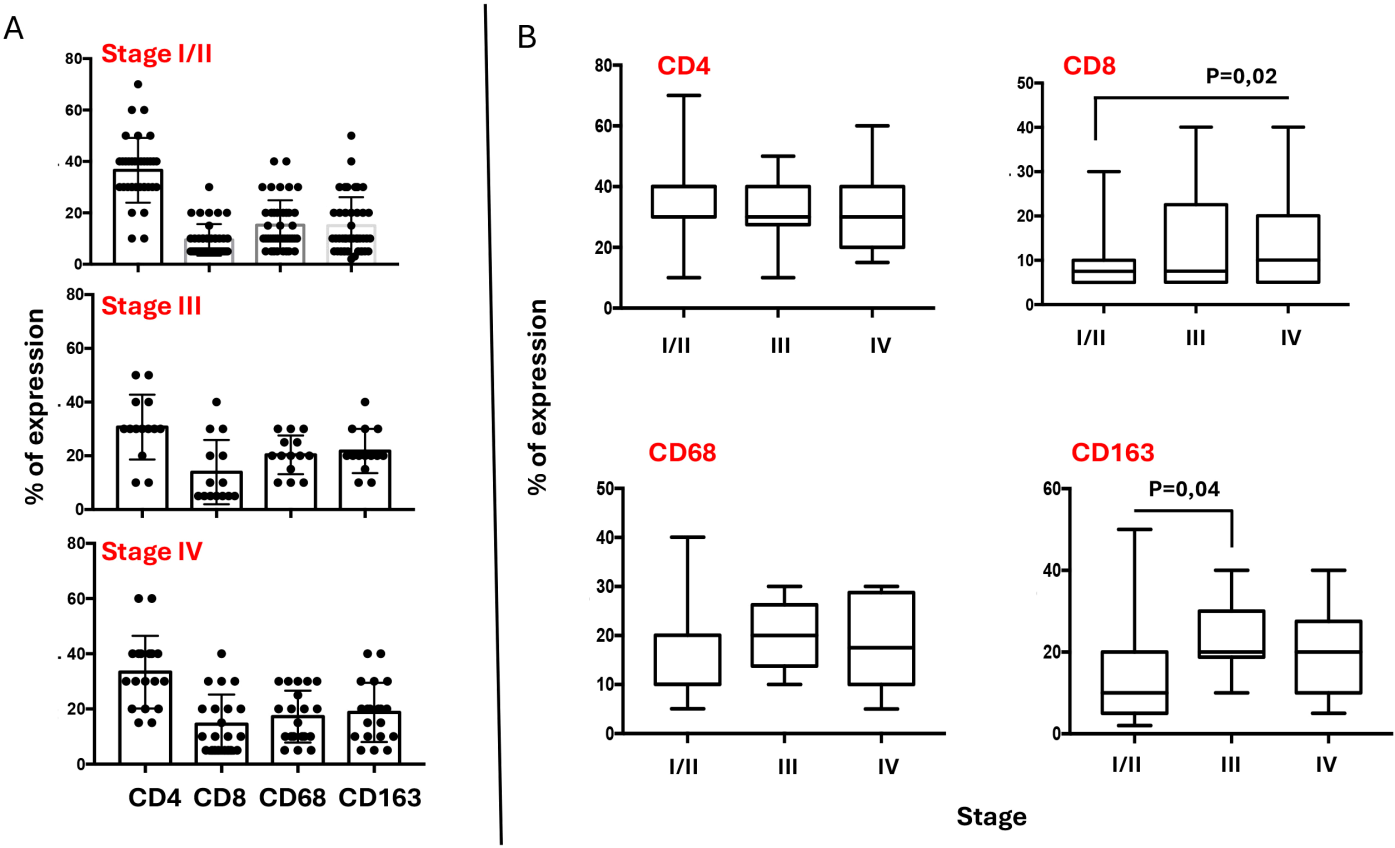
Composition of Inflammatory infiltrate across Ann Arbor stages. **(A)** Graphic representation of CD4, CD8, CD68 and CD163 densities in TME of HL lymph nodes, according to the stage. The percentage of each cellular component was quantified relative to the total lymph node cellularity, including both neoplastic cells and non-neoplastic cells. Some cell types may coexpress multiple lineage markers at varying levels. For statistical analysis see **Table 4**. **(B)** Variations of each immune cell subsets across the stages.

The inflammatory infiltrate in classical Hodgkin lymphoma is composed mainly of T cells (especially CD4 helper T cells, but also some CD8 cytotoxic T cells) and macrophages (23, 24). Additional components of the inflammatory infiltrate, which typically make up less than 5% each of the total cellular infiltrate, can include eosinophils, neutrophils, plasma cells, dendritic cells, mast cells, and fibroblasts. Though less abundant than T-cells, tumor-infiltrating macrophages play a pivotal role in supporting HRS survival. They are strongly associated with shortened survival in patients with classic Hodgkin’s lymphoma (25). We performed immunohistochemical examination of CD4, CD8, CD68, and CD163 subpopulations in tumor sections. Our finding confirms that the CD4^+^ T-cell component is significantly predominant among the inflammatory cells (**Figure 4A**). **Figure 4A** shows a graphical representation of the density of inflammatory cells across tumor stages, according to the Ann Arbor classification. CD4 T cells consistently represent the most abundant immune subset, with significantly higher counts than other populations (see **Table 4**), except in stage III, where the difference between CD4 and CD163 cell counts is not statistically significant. When comparing the densities of individual immune cell subpopulations across tumor stages, a progressive increase in CD8 cell numbers is observed with advancing stage (**Figure 4B**). This pattern suggests that CD8 T cells may be functionally ineffective. This hypothesis is consistent with the parallel increase in M2-like macrophages (i.e., CD163) (**Figure 4B**), which are likely to exert an inhibitory effect on CD8 cytotoxic activity, as we have previously demonstrated (26).

**Table 4:** Differences in subpopulation densities in inflammatory infiltrate according to Ann Arbor stage.

Stage	Tukey’s multiple comparisons test	Mean Diff,	95,00% CI of diff	Significant?	Summary	Adjusted P Value
	% CD4 vs. % CD8	27,1	20,94 to 33,26	Yes	****	<0,0001
	% CD4 vs. % CD68	21,33	15,28 to 27,38	Yes	****	<0,0001
I/II	% CD4 vs. % CD163	21,57	15,52 to 27,62	Yes	****	<0,0001
	% CD8 vs. % CD68	-5,77	-11,69 to 0,1497	No	ns	0,059
	% CD8 vs. % CD163	-5,526	-11,45 to 0,3936	No	ns	0,0767
	% CD68 vs. % CD163	0,2439	-5,563 to 6,05	No	ns	0,9995
	% CD4 vs. % CD8	16,79	6,653 to 26,92	Yes	***	0,0003
	% CD4 vs. % CD68	10,36	0,2245 to 20,49	Yes	*	0,0434
III	% CD4 vs. % CD163	8,929	-1,204 to 19,06	No	ns	0,1024
	% CD8 vs. % CD68	-6,429	-16,56 to 3,704	No	ns	0,3424
	% CD8 vs. % CD163	-7,857	-17,99 to 2,276	No	ns	0,1806
	% CD68 vs. % CD163	-1,429	-11,56 to 8,704	No	ns	0,9819
	% CD4 vs. % CD8	18,81	9,506 to 28,11	Yes	****	<0,0001
	% CD4 vs. % CD68	16,08	6,673 to 25,49	Yes	***	0,0001
IV	% CD4 vs. % CD163	14,58	5,173 to 23,99	Yes	***	0,0006
	% CD8 vs. % CD68	-2,726	-11,78 to 6,323	No	ns	0,8581
	% CD8 vs. % CD163	-4,226	-13,28 to 4,823	No	ns	0,6117
	% CD68 vs. % CD163	-1,5	-10,66 to 7,659	No	ns	0,9731

### 3.5 Stage-specific prognostic significance of TME subsets and FKBP51 expression in HL

**Figure 5:**
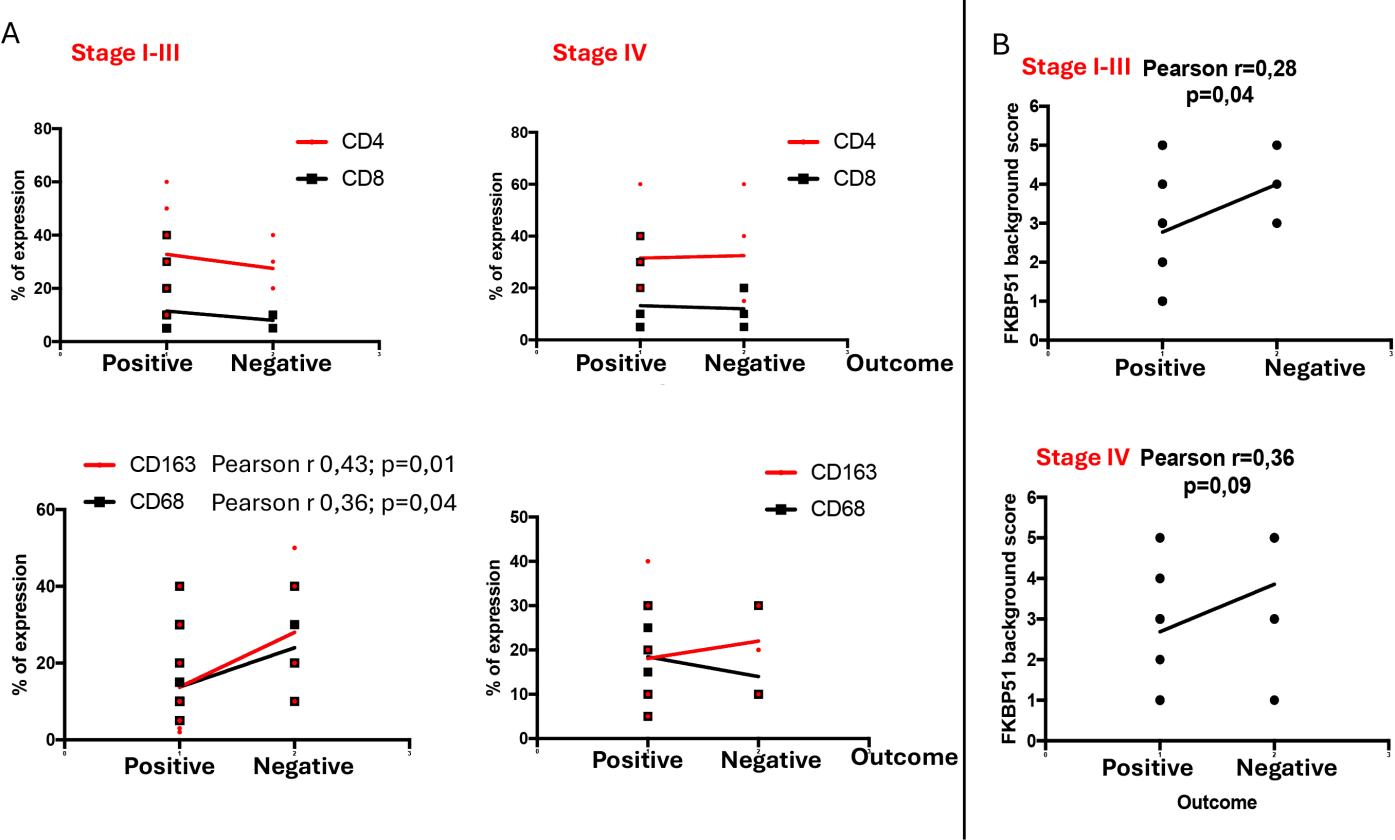
Stage-specific prognostic significance of TME subsets **(A)** and FKBP51 expression **(B)** in HL. Stages I, II, and III were grouped together and analyzed separately from stage IV. In stages I–III, CD68 and CD163 macrophages demonstrated prognostic relevance, along with FKBP51 expression.

To assess the prognostic value of each inflammatory subset within the TME, we grouped stages I, II, and III together and analyzed them separately from stage IV cases. Unfortunately, due to scarce prognostic information for stage I and II patients, we were unable to evaluate these stages separated from those of stage III. Our results indicate that only the macrophage subsets, specifically CD68 and CD163 cells, held prognostic value, being associated with poorer outcomes in stages I–III and not stage IV (**Figure 5A**). In contrast, CD4 T cells, despite being more abundant, did not impact prognosis (**Figure 5A**).

Using the same stage grouping, we analyzed the prognostic value of FKBP51 expression and found that FKBP51 retains its prognostic significance in stages I–III, whereas this significance is lost in stage IV disease (**Figure 5B**).

**Figure 6:**
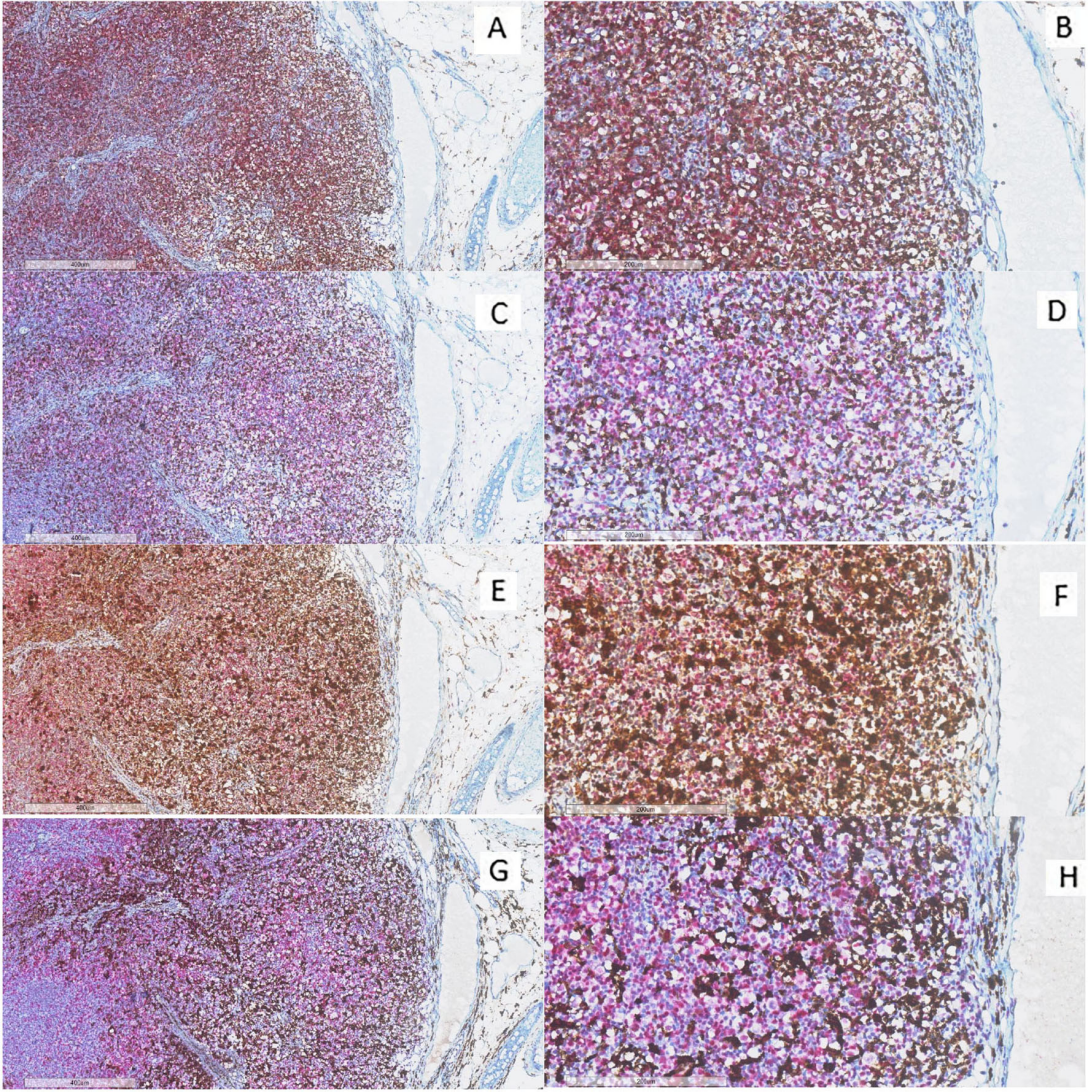
Immunohistochemical analysis of immune infiltrate. **(A, B)** A diffuse co-expression of CD4 and FKBP51 was observed in T-helper lymphocytes, resulting in a merged color (double staining for CD4, brown; and FKBP51, red; **(A)** original magnification, x6; **(B)** original magnification, x20). **(C, D)** A merged staining for CD8 and FKBP51 was seen in a minority of T-cytotoxic lymphocytes, with many of these cells being negative for FKBP51 (double staining for CD8, brown; and FKBP51, red; **(C)** original magnification, x6; **(D)** original magnification, x20). **(E, F)** a merged staining for CD68 and FKBP51 was observed in a minority of histiocytes, a large part of these cells is negative for FKBP51 (double staining for CD68, brown; and FKBP51, red; **(C)** original magnification, x6; **(D)** original magnification, x20). **(G, H)** A merged staining for CD163 and FKBP51 was noted in a minority of histiocytes, in which a large portion of the cells were negative for FKBP51 (double staining for CD163, brown; and FKBP51, red; **(G)** original magnification, x6; **(H)** original magnification, x20).

**Figure 7:**
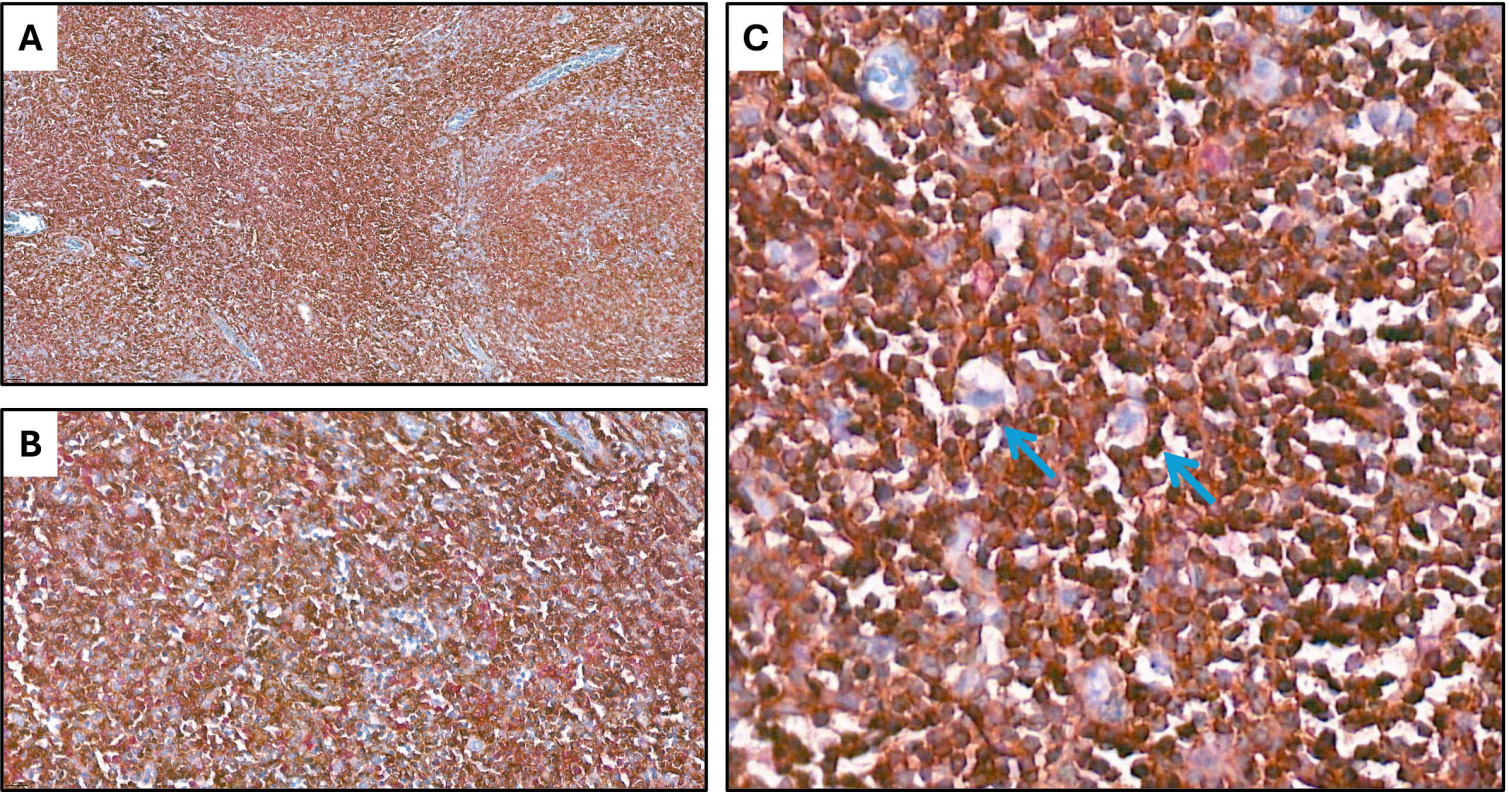
Co-expression of CD4 and FKBP51 **(A–C)** Double immunohistochemical staining demonstrates co-expression of CD4 (brown) and FKBP51 (red) in many T-helper lymphocytes, resulting in an overlapping color. The blue coloration reflects hematoxylin-stained nuclei in regions lacking marker expression. Notably, at the highest magnification, T-helper lymphocytes forming rosettes with HRS cells are diffusely and strongly reactive for FKBP51 (double staining for CD4 and FKBP51; **(A)** original magnification, x10; **(B)** original magnification, x40; **(C)** original magnification, x63; blue arrows highlighted the T-lymphocytes rosettes).

Double immunohistochemical staining has been performed, respectively, for CD4/FKBP51 (**Figures 6A, B**), CD8/FKBP51 (**Figures 6C, D**), CD68/FKBP51 (**Figures 6E, F**), and CD163/FKBP51 (**Figures 6G, H**), with red staining for FKBP51 and brown staining for the other. As we observed, all the T-helper lymphocytes co-express red staining for FKBP51 and brown staining for CD4, resulting in a merged color that we can identify as a bronze color (**Figures 6A, B**). This overlapping can be noted in all the fields, with the same intensity. Otherwise, T-cytotoxic lymphocytes express only brown staining for CD8, underlining the differences from T-helper lymphocytes (**Figures 6C, D**). In fact, in this case, we can see both the colors identifying the two different lymphocytic populations. We also noticed that the CD68 histiocytes closer to the HRS cells express more FKBP51(resulting in a merged staining bronze color) than histiocytes far from HRS cells that appear stained only brown for CD68 (**Figures 6E, F**). CD163 histiocytes are, in the majority of cases, negative for FKBP51, expressing only the brown staining for CD163 (**Figures 6G, H**). **Supplementary Figure S3** shows single IHC performed on consecutive serial sections, in parallel with the double IHC, using the conventional DAB chromogen (brown). At the highest magnification, T-helper lymphocytes forming rosettes with HRS cells are strongly reactive for FKBP51 (**Figure 7**).

**Figure 8:**
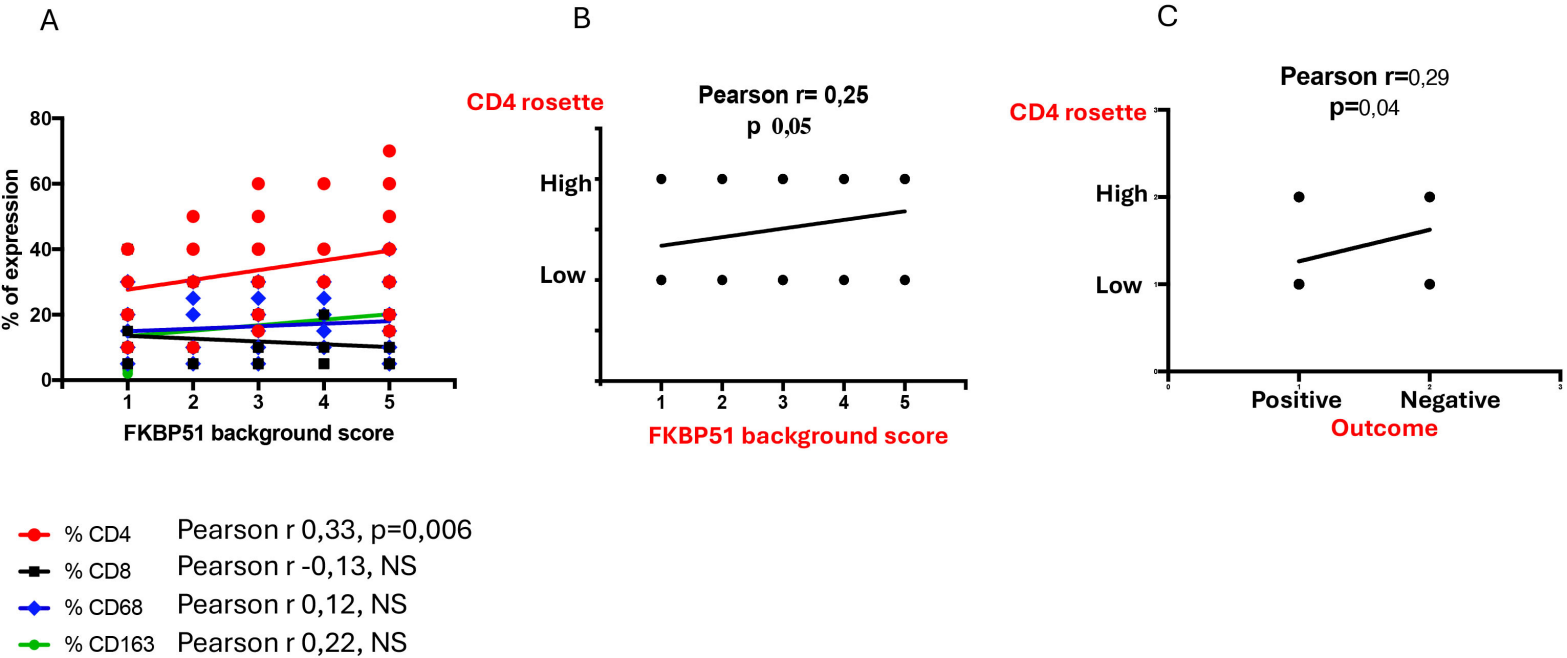
**(A)** Correlation analysis between the counts of TME subsets and FKBP51 background score: a linear relationship subsists between CD4 cell density and FKBP51 score. A high rosette score is significantly associated with increased FKBP51 expression **(B)** as well as with poorer patient prognosis **(C)**.

This finding shows that it was CD4 T cells and not the macrophages, despite their prognostic value, that exhibit the strongest FKBP51 expression. A correlation analysis between the counts of TME subsets and FKBP51 background score revealed a linear relationship between CD4 cell density and FKBP51 expression (**Figure 8A**). Notably, in HL, immune cells exhibit varied spatial distributions within the TME, interstitial, nodular, or diffuse. Among these, CD4^+^ T cells uniquely form a characteristic rosette-like arrangement around tumor cells. The rosette pattern was evaluated using a histological scoring system (high vs. low), supported by image analysis to quantify both rosette density and extent. A high rosette score was significantly associated with increased FKBP51 expression (**Figure 8B**) as well as with poorer patient prognosis (**Figure 8C**), suggesting that the spatial architecture of the immune infiltrate may hold prognostic relevance in Hodgkin lymphoma.

### 3.6 Gene expression profiles of tumor biopsies reveal an FKBP51 association with proliferative and anti-apoptosis markers

**Figure 9:**
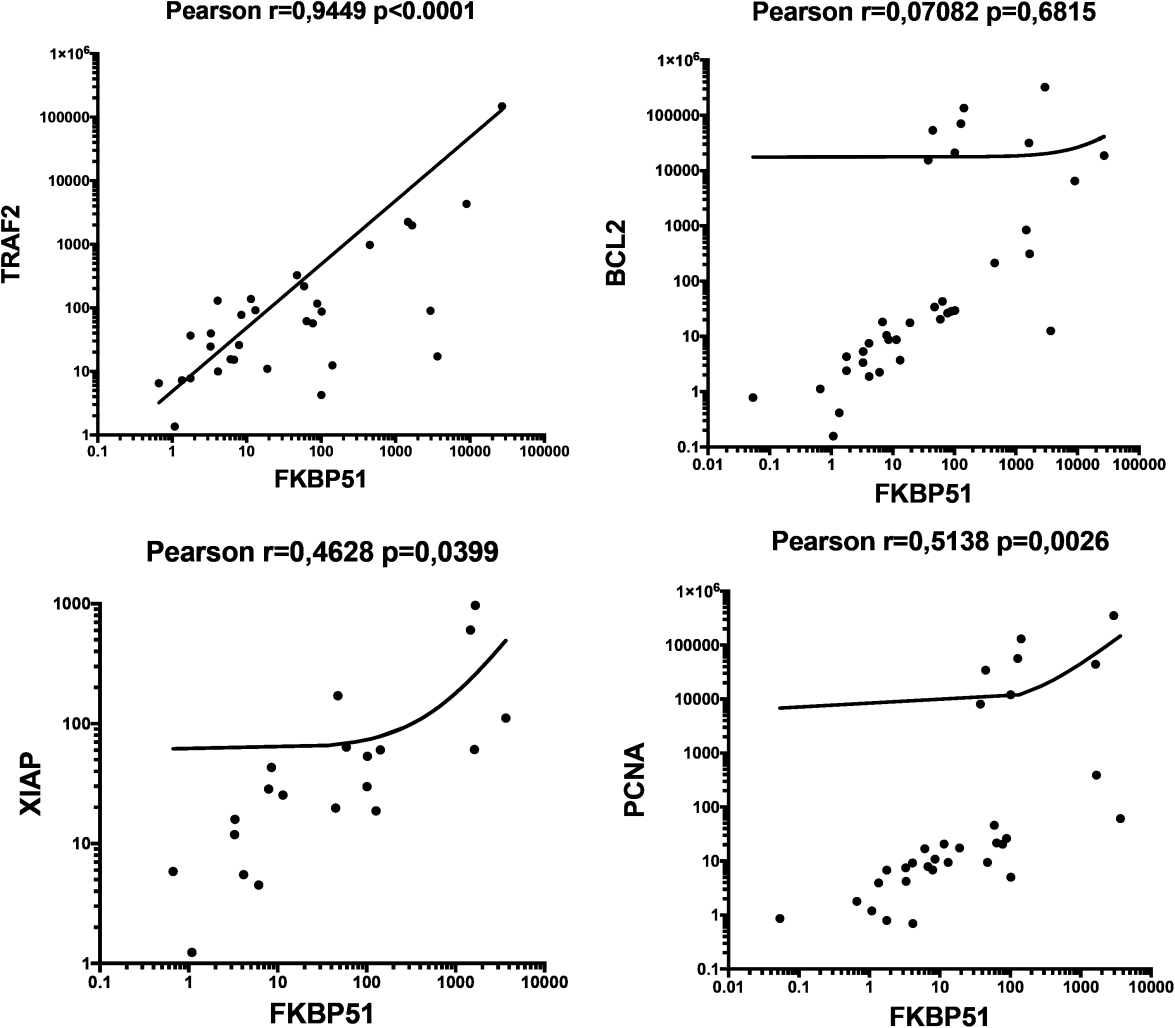
Scatterplots of Pearson’s correlations between *FKBP5* and *TRAF2, BCL2, XIAP and PCNA*, gene transcripts, measured by qPCR. The total numbers of cases analyzed were as follows: 36, for *FKBP5* and *BCL2; 31, for TRAF2; 20 for XIAP; 32*, for *PCNA*.

H/RS cells typically make up only a minority (<1–2%) of the tumor mass. Their specific gene expression is underrepresented in bulk RNA (27). With the aim to gain more insights into the characteristics of the TME, we thought to analyze the transcript levels of *FKBP5* alongside various genes related to proliferation and apoptosis in RNA extracted from tumor biopsies. *PCNA, BCL2, TRAF2, XIAP* and *FKBP5* expression levels in whole RNAs extracted by 36 tumor tissue samples were quantified as relative expression, using expression from healthy donor PBMCs as a reference sample (=1). Although, with high variability, most transcript levels were quantifiable in tumor tissue samples. **Figure 9** shows a correlative analysis of gene expression. *FKBP51* was associated with *TRAF2*, an essential element in the activation of NF-κB signaling pathway (28), and with *XIAP* and *PCNA*, suggesting an active NF-κB/anti-apoptosis/proliferative axis in the TME (**Figure 9**). Consistent with IHC results, no correlation was found between FKBP51 and Bcl2. The lack of correlation between *BCL2* and *TRAF2* suggests that Bcl-2 is not activated by the canonical NF-κB pathway (see **Supplementary Figure S4**). This aligns with previous studies that have shown the dependence of Bcl-2 on non-canonical NF-κB pathway (29, 30). Furthermore, the significant correlation found between *TRAF2* and *XIAP* indicates that the canonical NF-κB pathway concurs in the anti-apoptotic mechanisms of CD4 T lymphocytes (Supplementary information, **Supplementary Figure S4**). Analyzing the expression levels of FKBP51, TRAF2, Bcl-2, XIAP, and PCNA transcripts in relation to the Ann Arbor stage, we observed that TRAF2 positively correlated with stage (Supplementary information, **Supplementary Figure S5**).

## 4 Discussion

Hodgkin lymphoma is a unique lymphoid malignancy characterized by a minority of neoplastic H/RS cells surrounded by a predominant population of non-neoplastic immune cells. These tumor-associated lymphoid cells play a fundamental role in disease biology and have important prognostic implications.

One of the key findings of our study is that FKBP51 expression in tumor-infiltrating lymphocytes acts as an independent adverse prognostic marker in HL. While FKBP51 was also detected in H/RS cells, predominantly within the nucleus, its expression in tumor cells was not associated with clinical outcome. In contrast, FKBP51 expression in the surrounding lymphoid background correlated significantly with poorer prognosis and remained significant in multivariate analysis. These data point to a functional role of the TME, rather than the tumor cells alone, in disease progression. The differential expression pattern of FKBP51 may reflect distinct NF-κB regulatory mechanisms. In H/RS cells, recurrent inactivating mutations in NFKBIA (31), which encodes the IκBα inhibitor, result in constitutive NF-κB activation that is thus disconnected from both positive and negative regulators (31).

Immunohistochemical characterization confirmed that CD4 T cells represent the predominant immune subset within the TME, followed by CD8 T cells and macrophages (CD68 and CD163). Despite their abundance, CD4 T cells did not display prognostic impact. Double immunostaining revealed that CD4 T cells are the main FKBP51-expressing population, with staining intensity correlating with their spatial proximity to H/RS cells. FKBP51 is known to enhance immune activity (15) via NF-κB (14) and Akt (32) signaling pathways, making its association with poor prognosis somewhat unexpected. Nonetheless, the strong FKBP51 immunoreactivity observed in CD4^+^ cells is indicative of an immunologically active phenotype, potentially engaged in sustained helper functions.

In contrast, CD8 T cells show relatively low FKBP51 expression and are less prominent, particularly in early-stage disease. Interestingly, their numbers increased in advanced stages, possibly reflecting a compensatory, yet ultimately ineffective, antitumor response. This functional inefficacy may be explained by the concurrent accumulation of tumor-associated macrophages. In previous work, we demonstrated that CD163 macrophages can profoundly inhibit CD8 T cell cytotoxicity in co-culture systems (26), reinforcing their role as potent immunosuppressive players within the HL microenvironment (25).

The paradox of highly immunoreactive CD4 T cells alongside hyporeactive CD8 T cells can be reconciled by considering the spatial architecture of the TME in HL lymph node. A key factor modulating immune interactions is the physical organization of immune subsets. CD4 T cells are frequently arranged in rosette-like structures tightly encircling H/RS cells, likely creating a protective niche that favors tumor survival (9, 11, 23, 33). In contrast, CD8 T cells are more diffusely distributed throughout the lymph node, as are TAMs, which virtually exert broader immunosuppressive effects that systemically limit CD8 effector function. This spatial dichotomy enables CD4 T cells to act as functional allies of the tumor, delivering trophic and immunomodulatory cues, while TAMs maintain a suppressive environment that hinders effective cytotoxic responses.

Gene expression profiling further indicated that FKBP51 is significantly associated with transcripts involved in proliferation (PCNA) and apoptosis resistance (TRAF2, XIAP), underscoring its potential role in promoting a protumor, NF-κB-dependent phenotype in support of H/RS cells. Notably, TRAF2 expression is the only transcript among those analyzed to correlate positively with disease stage, reinforcing its relevance in disease progression.

### 4.1 Limitations

While using bulk HL biopsies offers a more comprehensive understanding of gene expression compared to single-cell analysis, single-cell RNA sequencing could be used to better profile H/RS cells separately from the TME, potentially leading to more thorough conclusions. An even more effective approach is spatial transcriptomics that preserves tissue architecture while providing information about the physical interactions and proximity between cells. This technology should greatly aid future studies to enhance our understanding of how interactions between cancer cells, CD4 T cells, and other immune or stromal populations influence disease pathology.

The correlation of FKBP51 between lymphocytes and tumor cells is in line with the notion of a cell-cell connection. However, FKBP51 expression in H/RS cells is not linked to prognosis, leaving its role in H/RS cells unclear.

Further research is needed to explore all these aspects more thoroughly.

## 5 Conclusion

Our study highlights prognostic relevance of macrophages in early-stage disease, in accordance with previous study and FKBP51 as part of a proliferative and anti-apoptotic transcriptional network. Its expression pattern, particularly in the context of spatial architecture such as the rosette-like CD4 arrangement, could serve as a histopathological biomarker to improve risk stratification (34–36).

## Data Availability

The datasets presented in this study can be found in online repositories. The names of the repository/repositories and accession number(s) can be found in the article/**Supplementary Material**, and at the link below: https://github.com/repositoryromano/doi-10.3389-fimmu.2025.1604920.

## Supplementary material

Supplementary Figure 1 Immunohistochemical analysis of Reed Sternberg cell. **(A)** Double immunostaining for CD30 (red) and FKBP51 (brown) (magnification 20X). **(B)** Single immunostaining for CD30 (brown) (magnification 20X). **(C)** Single immunostaining for FKBP51 (brown) (magnification 20X).

Supplementary Figure 2 Kaplan–Meier survival curves based on H/RS Bcl-2 and background FKBP51 expression. Patients were stratified according to immunohistochemical expression levels of Bcl-2 (left) and FKBP51 (right). Survival outcomes were compared using the Gehan–Breslow–Wilcoxon test.

Supplementary Figure 3 Multiple 4-μm-thick serial sections were cut from a single formalin-fixed, paraffin-embedded tissue block. The first five serial sections were used for single immunostaining of the following markers: FKBP51 (BROWN), CD4 (BROWN), CD8 (BROWN), CD68 (BROWN), and CD163 (BROWN). The subsequent four sections (sixth to ninth) were employed for double immunostaining with the following combinations: CD4 (BROWN) and FKBP51 (RED); CD8 (BROWN) and FKBP51 (RED); CD68 (BROWN) and FKBP51 (RED); and CD163 (BROWN) and FKBP51 (RED). The resulting images have been assembled as follows: **(A)** Double immunostaining for CD4 (BROWN) and FKBP51 (RED); **(B)** Single immunostaining for CD4 (BROWN); **(C)** Single immunostaining for FKBP51 (BROWN); **(D)** Double immunostaining for CD8 (BROWN) and FKBP51 (RED); **(E)** Single immunostaining for CD8 (BROWN); **(F)** Double immunostaining for CD68 (BROWN) and FKBP51 (RED); **(G)** Single immunostaining for CD68 (BROWN); **(H)** Double immunostaining for CD163 (BROWN) and FKBP51 (RED); **(I)** Single immunostaining for CD163 (BROWN).

Supplementary Figure 4 Scatterplots of Pearson’s correlations between *TRAF2 and BCL2 or XIAP* gene transcripts, measured by qPCR.

Supplementary Figure 5 Linear correlation between TRAF2 transcript levels and stages according to Ann Arbor.
