# Supplementary material for: Expression and prognostic value of FKBP51 in Hodgkin lymphoma
Source: Front Immunol. 2025 Nov 3;16:1604920. doi: 10.3389/fimmu.2025.1604920 (PMC12620377; doi:10.3389/fimmu.2025.1604920)
Supplement: Supplementary Figure 1 — Immunohistochemical analysis of Reed Sternberg cell. (A) Double immunostaining for CD30 (red) and FKBP51 (brown) (magnification 20X). (B) Single immunostaining for CD30 (brown) (magnification 20X). (C) Single immunostaining for FKBP51 (brown) (magnification 20X). [file DataSheet1.docx]

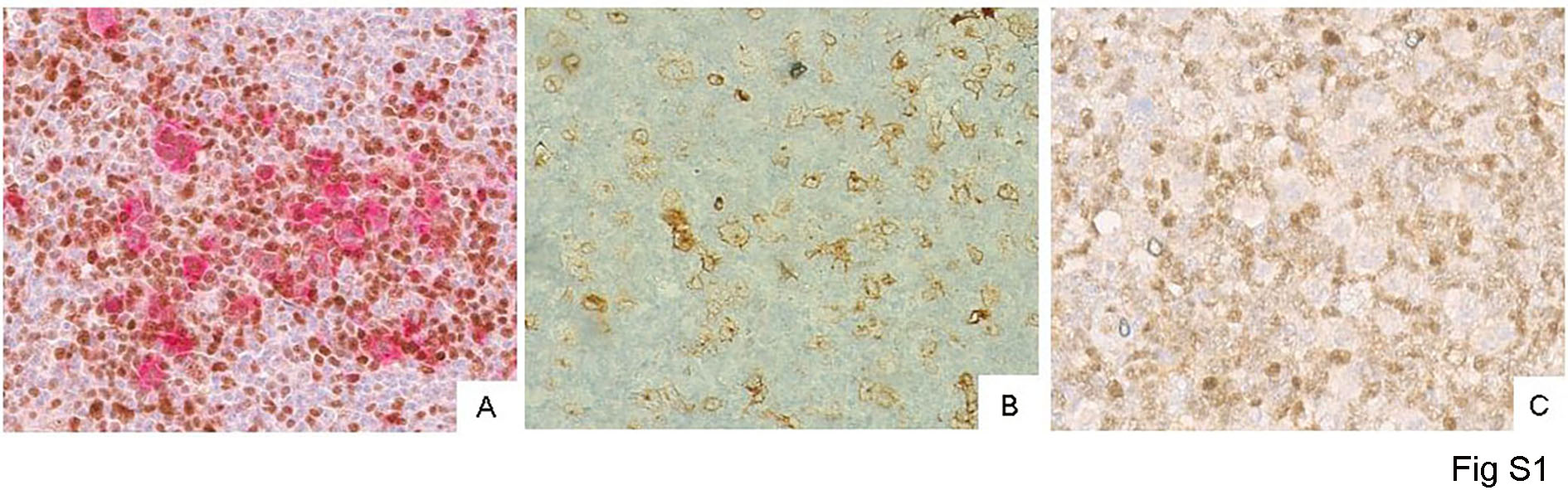


**Figure S1**: Immunohistochemical analysis of Reed Sternberg cell. A) Double immunostaining for CD30 (red) and FKBP51 (brown) (magnification 20X). B) Single immunostaining for CD30 (brown) (magnification 20X). C) Single immunostaining for FKBP51 (brown) (magnification 20X).
