# Supplementary material for: Expression and prognostic value of FKBP51 in Hodgkin lymphoma
Source: Front Immunol. 2025 Nov 3;16:1604920. doi: 10.3389/fimmu.2025.1604920 (PMC12620377; doi:10.3389/fimmu.2025.1604920)
Supplement: Supplementary Figure 2 — Kaplan–Meier survival curves based on H/RS Bcl-2 and background FKBP51 expression. Patients were stratified according to immunohistochemical expression levels of Bcl-2 (left) and FKBP51 (right). Survival outcomes were compared using the Gehan–Breslow–Wilcoxon test. [file DataSheet2.docx]

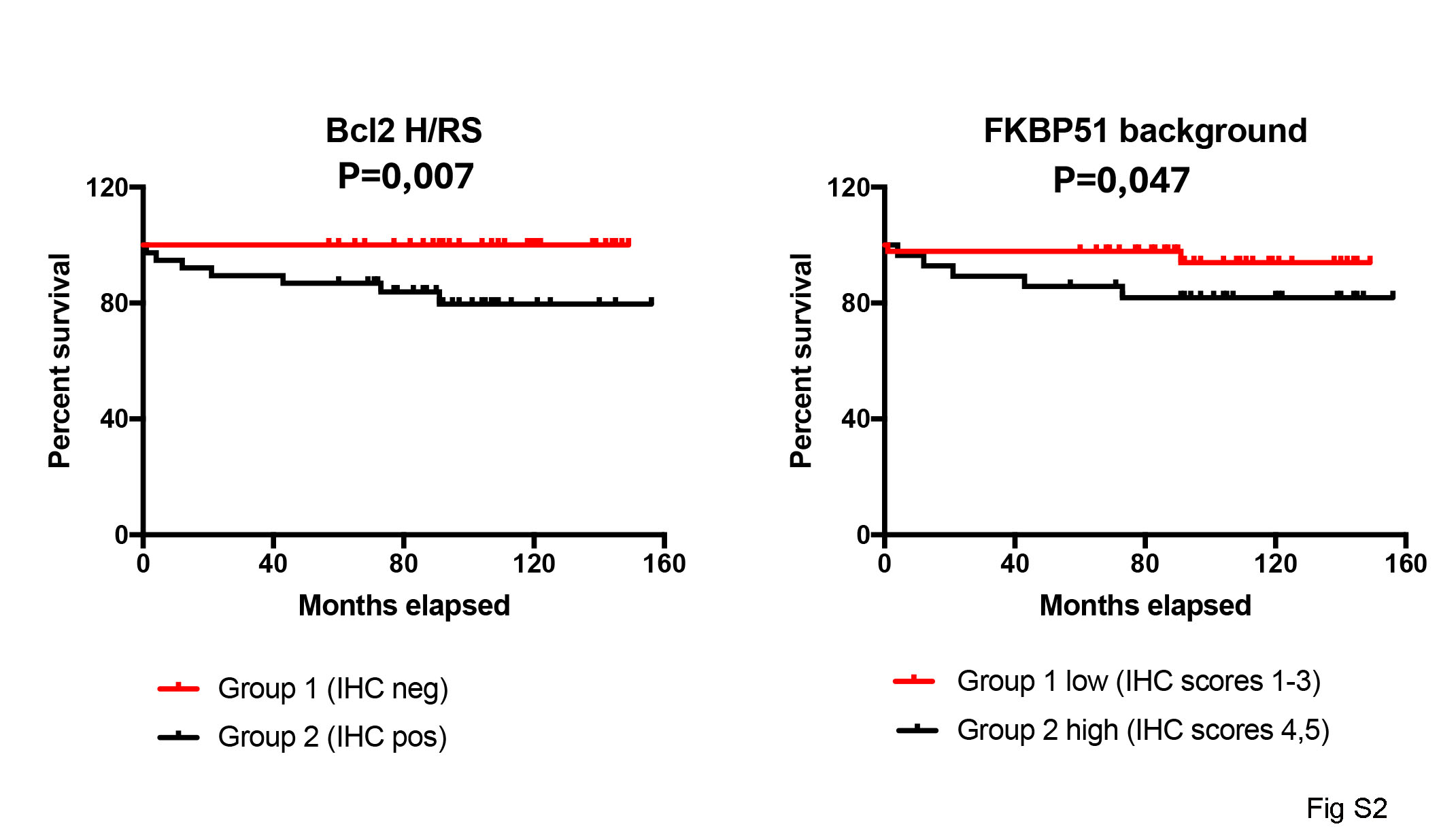


**Figure S2**: Kaplan–Meier survival curves based on H/RS Bcl-2 and background FKBP51 expression. Patients were stratified according to immunohistochemical expression levels of Bcl-2 (left) and FKBP51 (right). Survival outcomes were compared using the Gehan–Breslow–Wilcoxon test.
