# Supplementary material for: Expression and prognostic value of FKBP51 in Hodgkin lymphoma
Source: Front Immunol. 2025 Nov 3;16:1604920. doi: 10.3389/fimmu.2025.1604920 (PMC12620377; doi:10.3389/fimmu.2025.1604920)
Supplement: Supplementary Figure 3 — Multiple 4-μm-thick serial sections were cut from a single formalin-fixed, paraffin-embedded tissue block. The first five serial sections were used for single immunostaining of the following markers: FKBP51 (BROWN), CD4 (BROWN), CD8 (BROWN), CD68 (BROWN), and CD163 (BROWN). The subsequent four sections (sixth to ninth) were employed for double immunostaining with the following combinations: CD4 (BROWN) and FKBP51 (RED); CD8 (BROWN) and FKBP51 (RED); CD68 (BROWN) and FKBP51 (RED); and CD163 (BROWN) and FKBP51 (RED). The resulting images have been assembled as follows: (A) Double immunostaining for CD4 (BROWN) and FKBP51 (RED); (B) Single immunostaining for CD4 (BROWN); (C) Single immunostaining for FKBP51 (BROWN); (D) Double immunostaining for CD8 (BROWN) and FKBP51 (RED); (E) Single immunostaining for CD8 (BROWN); (F) Double immunostaining for CD68 (BROWN) and FKBP51 (RED); (G) Single immunostaining for CD68 (BROWN); (H) Double immunostaining for CD163 (BROWN) and FKBP51 (RED); (I) Single immunostaining for CD163 (BROWN). [file DataSheet3.docx]

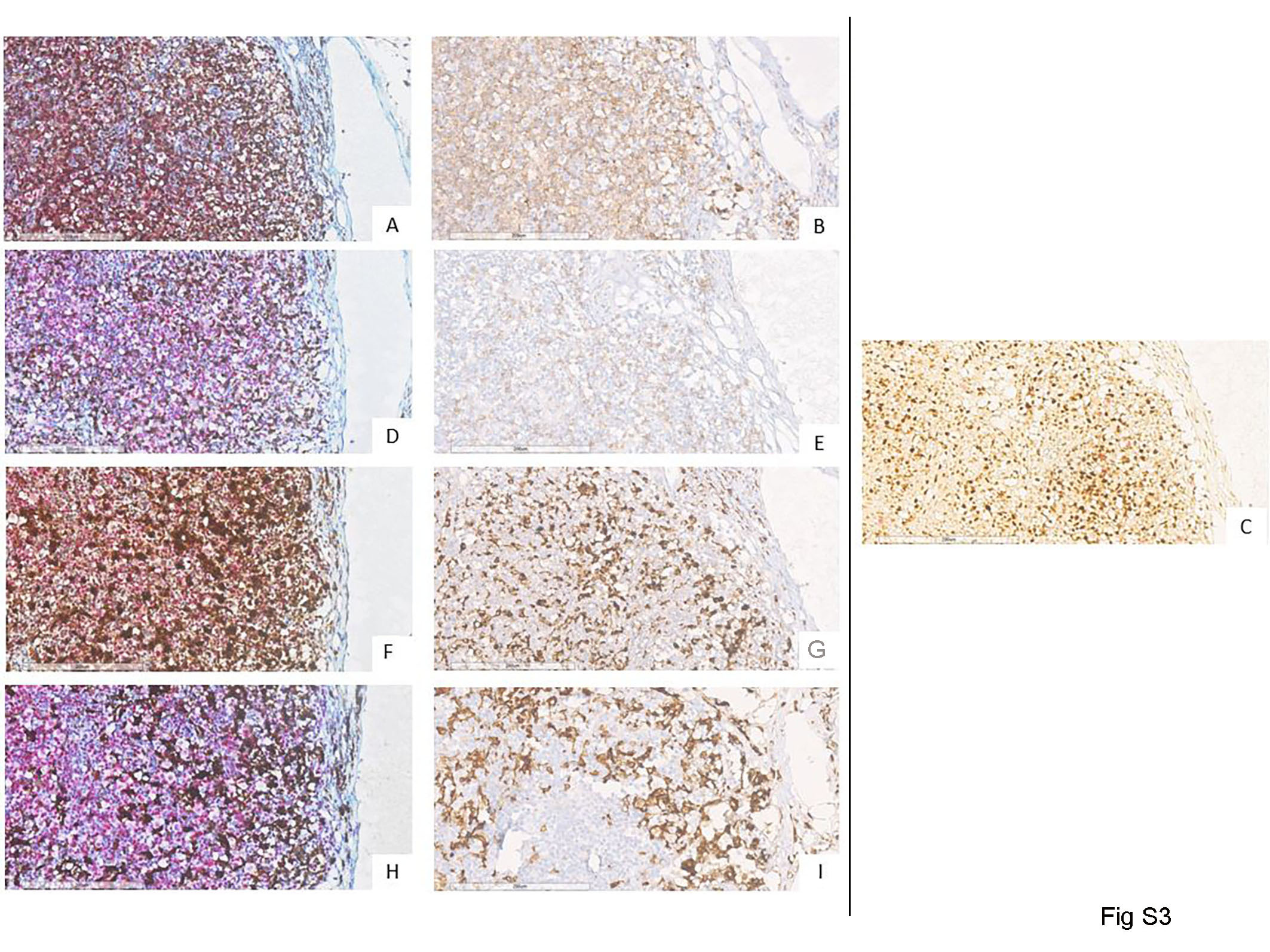


**Figure S3**: Multiple 4-μm-thick serial sections were cut from a single formalin-fixed, paraffin-embedded tissue block. The first five serial sections were used for single immunostaining of the following markers: FKBP51 (BROWN), CD4 (BROWN), CD8 (BROWN), CD68 (BROWN), and CD163 (BROWN). The subsequent four sections (sixth to ninth) were employed for double immunostaining with the following combinations: CD4 (BROWN) and FKBP51 (RED); CD8 (BROWN) and FKBP51 (RED); CD68 (BROWN) and FKBP51 (RED); and CD163 (BROWN) and FKBP51 (RED). The resulting images have been assembled as follows:

**A)** Double immunostaining for CD4 (BROWN) and FKBP51 (RED);
**B)** Single immunostaining for CD4 (BROWN);
**C)** Single immunostaining for FKBP51 (BROWN);
**D)** Double immunostaining for CD8 (BROWN) and FKBP51 (RED);
**E)** Single immunostaining for CD8 (BROWN);
**F)** Double immunostaining for CD68 (BROWN) and FKBP51 (RED);

**G)** Single immunostaining for CD68 (BROWN);
**H)** Double immunostaining for CD163 (BROWN) and FKBP51 (RED);
**I)** Single immunostaining for CD163 (BROWN);
