# Supplementary material for: Expression and prognostic value of FKBP51 in Hodgkin lymphoma
Source: Front Immunol. 2025 Nov 3;16:1604920. doi: 10.3389/fimmu.2025.1604920 (PMC12620377; doi:10.3389/fimmu.2025.1604920)
Supplement: Supplementary Figure 4 — Scatterplots of Pearson’s correlations between TRAF2 and BCL2 or XIAP gene transcripts, measured by qPCR. [file DataSheet4.docx]

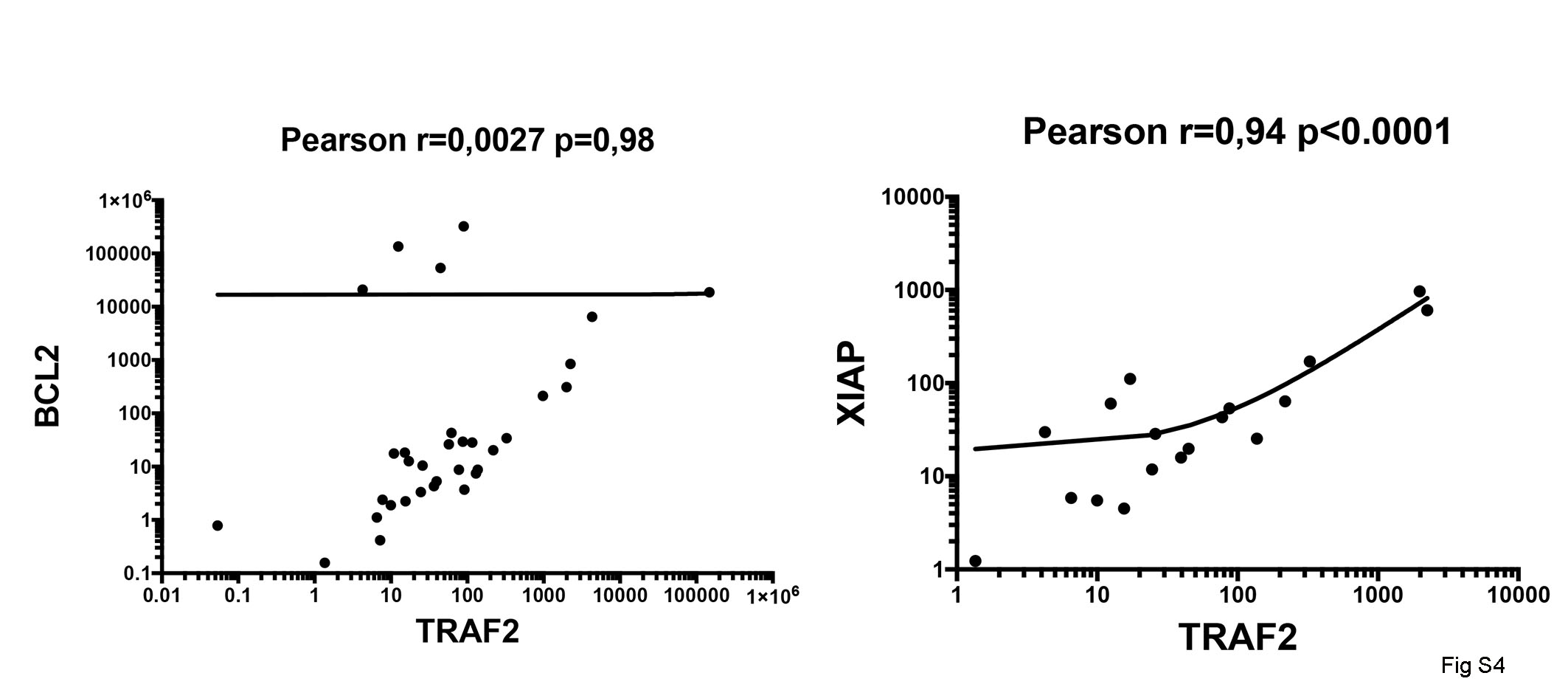


**Figure S4**: Scatterplots of Pearson’s correlations between TRAF2 and BCL2 or XIAP gene transcripts, measured by qPCR.
