# Supplementary figures and images for: Expression and prognostic value of FKBP51 in Hodgkin lymphoma
Source: Front Immunol. 2025 Nov 3;16:1604920. doi: 10.3389/fimmu.2025.1604920 (PMC12620377; doi:10.3389/fimmu.2025.1604920)

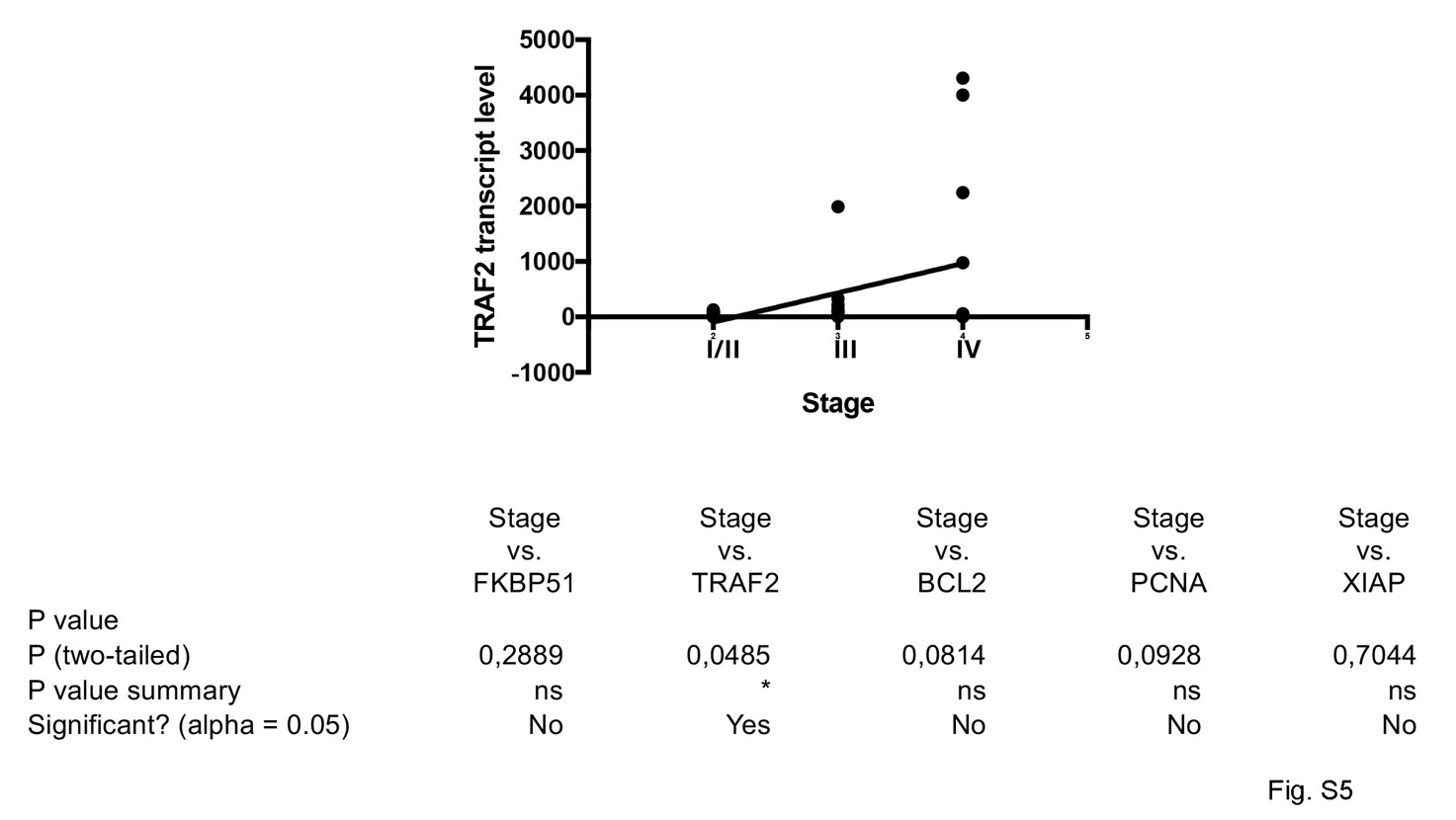


**Figure S5**: Linear correlation between TRAF2 transcript levels and stages according to Ann Arbor.

Supplement: Supplementary Figure 5 — Linear correlation between TRAF2 transcript levels and stages according to Ann Arbor. [file DataSheet5.docx]
